# The Antimicrobial Activity of Curcumin and Xanthohumol on Bacterial Biofilms Developed over Dental Implant Surfaces

**DOI:** 10.3390/ijms24032335

**Published:** 2023-01-25

**Authors:** Andrea Alonso-Español, Enrique Bravo, Honorato Ribeiro-Vidal, Leire Virto, David Herrera, Bettina Alonso, Mariano Sanz

**Affiliations:** 1 ETEP (Etiology and Therapy of Periodontal and Peri-Implant Diseases) Research Group, Faculty of Dentistry, Complutense University, 28040 Madrid, Spain; 2 Department of Periodontology, Faculty of Dentistry, University of Porto, 4200-393 Porto, Portugal; 3 Department of Anatomy and Embryology, Faculty of Optics, Complutense University, 28040 Madrid, Spain

**Keywords:** curcumin, xanthohumol, peri-implantitis, oral biofilms, scanning electron microscopy, confocal laser microscopy, polymerase chain reaction, in vitro, antibacterial, antibiofilm

## Abstract

In search for natural products with antimicrobial properties for use in the prevention and treatment of peri-implantitis, the purpose of this investigation was to evaluate the antimicrobial activity of curcumin and xanthohumol, using an in vitro multi-species dynamic biofilm model including *Streptococcus oralis, Actinomyces naeslundii, Veillonella parvula, Fusobacterium nucleatum, Porphyromonas gingivalis* and *Aggregatibacter actinomycetemcomitans*. The antimicrobial activities of curcumin (5 mM) and xanthohumol (100 μM) extracts, and the respective controls, were evaluated with 72-h biofilms formed over dental implants by their submersion for 60 seconds. The evaluation was assessed by quantitative polymerase chain reaction (qPCR), confocal laser scanning microscopy (CLSM) and scanning electron microscopy (SEM). For the data analysis, comparisons were tested applying ANOVA tests with *post-hoc* Bonferroni corrections to evaluate the antimicrobial activity of both extracts. With qPCR, statistically significant reductions in bacterial counts were observed for curcumin and xanthohumol, when compared to the negative control. The results with CLSM and SEM were consistent with those reported with qPCR. It was concluded that both curcumin and xanthohumol have demonstrated antimicrobial activity against the six bacterial species included in the dynamic in vitro biofilm model used.

## 1. Introduction

Rehabilitation with dental implants is considered the gold standard for the treatment of full and partial edentulism. High percentages of success for osseointegration and survival have been reported, but peri-implant diseases have, nowadays, a huge impact. These diseases have been recently classified in the 2018 classification of the American Academy of Periodontology (AAP) and the European Federation of Periodontology (EFP) as peri-implant mucositis and peri-implantitis [1], and peri-implantitis may affect 20% of patients with implant-supported restorations [2].

The etiology of peri-implant diseases is associated with the inflammatory reaction of the peri-implant tissues to the accumulation of bacterial biofilms at the implant surface or at the restorative components [3,4]. Thus, biofilm control is crucial in their management, either mechanically or chemically, to arrest/control inflammation and to reestablish peri-implant health, when combined with effective oral hygiene practices [5,6].

The mechanical decontamination of implant surfaces has been proven effective in eliminating calculus deposits and residual debris; however, the presence of the implant threads, grooves and the moderately rough surface topography of most currently used dental implants, makes this decontamination process usually ineffective to fully eliminate these biofilms [7]. As a consequence, the adjunctive use of chemical agents has been advocated to supplement the mechanical measures on the implant surface, and related surfaces biofilm decontamination process, either through their bactericidal activity, or through their impact in reducing the organic components of the bacteria, or the destruction of their endotoxins [8,9,10,11,12,13,14]. 

In the search for the ideal antimicrobial agents to decontaminate implant and related surfaces, several antiseptic agents have been tested in the laboratory, mainly using in vitro biofilm models. Agents such as local and systemic antimicrobials [15,16,17], probiotics [18], postbiotics [19,20] or photodynamic therapy [21] have reported heterogeneous results and, currently, a gold standard treatment for implant surface decontamination has not been defined, since none of the aforementioned strategies have provided superior outcomes [12]. 

These results may be due to the lack of a well-validated in vitro multispecies biofilm model. Our research group has described and validated a dynamic multispecies in vitro biofilm model that allows the formation of predictable biofilms in different surface topographies, mimicking at the same time the fluid dynamics and environmental conditions met in the oral cavity [22,23]. 

Within this scenario, the advent of alternative antimicrobial agents, such as natural herbal agents, has gained attention worldwide [24,25,26]. Among these substances, **curcumin** (curcumin I, diferuloylmethane) a natural pigment, with a characteristic yellow–orange color, native to tropical South Asia, is usually found in the rhizomes of turmeric (*Curcuma longa* L.) belonging to the ginger family (*Zingiberaceae*) [27]. 

Although this extract has been historically used and advocated as a safe and effective treatment for a variety of diseases [28,29,30,31], it has gained attention in recent years due to the publication of numerous in vitro and in vivo studies confirming the health-promoting effects associated, primarily with its strong antioxidant and anti-inflammatory activities [32,33,34]. However, this natural extract also exhibits antibacterial, antifungal, antiviral, antiprotozoal, and antiparasitic properties [35,36,37]. 

**Figure 1:**
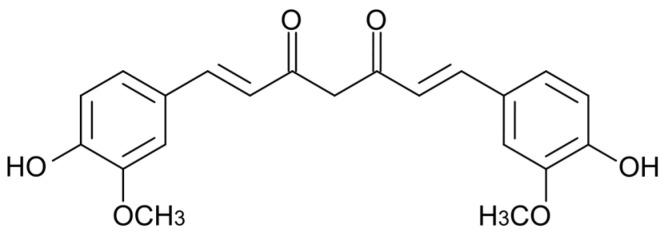
Chemical structure of curcumin.

This lipophilic polyphenol, with a chemical formula of C_21_H_20_O_6_ and a molecular weight of 368.38 g/mol, is a dimeric derivative of ferulic acid, composed of two *o*-methoxyphenol rings connected by a heptadienedione chain [27] (Figure 1). Curcumin is the principal bioactive compound of turmeric powder, which together with other essential oils and curcuminoids is contained in the oriental spice commonly obtained from this plant [38,39]. 

**Figure 2:**
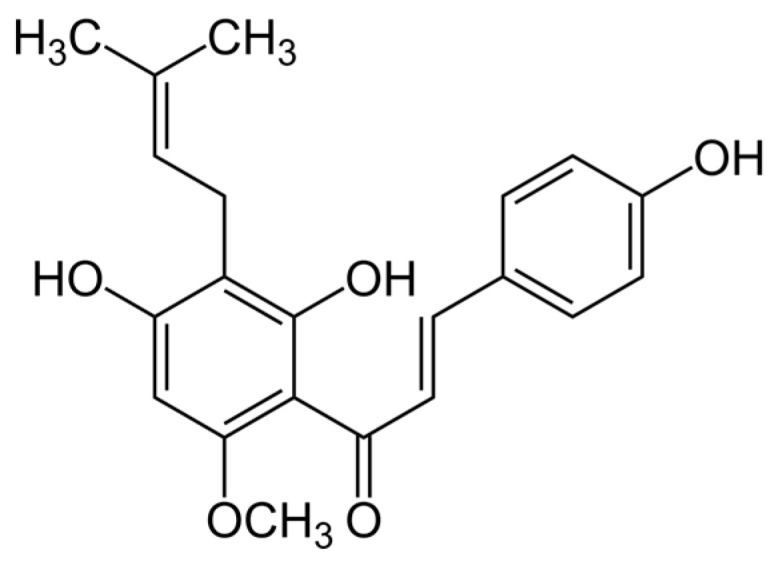
Chemical structure of xanthohumol.

Another similar substance, also known since ancient times, is **xanthohumol**, a derivative from the female flowers of the hops plant (*Humulus lupulus* L), which is a prenylated flavonoid (Figure 2) with potential antiseptic, anti-inflammatory, antidiuretic and antioxidant properties [40]. In 2013, the antibiofilm activity of hop-derived compounds was first published by Rozalski et al. [40].

These two substances, however, have not been previously studied for their effect on oral bacteria biofilms developed on implant surfaces [41,42,43,44]. It was, therefore, the purpose of this in vitro investigation, using a validated multispecies dynamic biofilm model, to evaluate the activity of curcumin and xanthohumol in the decontamination of dental implant surfaces [22,23].

## 2. Results

### 2.1. Antibacterial Effect of Curcumin and Xanthohumol on Planktonic Bacteria 

The following minimum inhibitory concentrations (MICs) and minimum bactericidal concentrations (MBCs) for xanthohumol and curcumin were determined against the six bacterial strains in planktonic state, as described in the Section 4:

(a) For xanthohumol, the MICs were established at 20 µM for Streptococcus oralis, Veillonella parvula, Actinomyces naeslundii and Fusobacterium nucleatum, 10 µM for Porphyromonas gingivalis and 100 µM for Aggregatibacter actinomycetemcomitans. The MBCs were established at 20 µM for A. naeslundii, V. parvula and F. nucleatum, 10 µM for P. gingivalis, 50 µM for S. oralis and 100 µM for A. actinomycetemcomitans.
(b) For curcumin, the MICs were established at 1 mM for S. oralis, A. naeslundii and A. actinomycetemcomitans and 500 µM for V. parvula, F. nucleatum and P. gingivalis and the MBCs at 1 mM for S. oralis, V. parvula, A. naeslundii, F. nucleatum and A. actinomycetemcomitans and 500 µM for P. gingivalis.

### 2.2. Antibacterial Effect of the Curcumin and Xanthohumol on the Dynamic Biofilm Model

#### 2.2.1. Quantitative Polymerase Chain Reaction (qPCR) Analysis 

The six tested bacterial species showed statistically significant reductions in bacterial counts (colony-forming units (CFUs) mL^−1^) after treatment with xanthohumol or curcumin (Table 1). By xanthohumol these reductions in cell viability amount to 97.47% for *S. oralis*, 96.64% for *A.* naeslundii, 98.52% for *V. parvula*, 95.90% for *F. nucleatum*, 99.20% for *P. gingivalis* and 96.64% for *A. actinomycetemcomitans*. With curcumin the reductions reached to 98.89% for *S. oralis*, 97.90% for *A.* naeslundii, 99.71% for *V. parvula*, 92.71% for *F. nucleatum*, 99.72% for *P. gingivalis* and 98.79% for *A. actinomycetemcomitans*, when compared to the phosphate-buffered saline (PBS) negative control, and statistically significant (Table 1 and Table 2).

Thus, the bactericidal effect of curcumin was slightly higher than that of xanthohumol and of chlorhexidine (CHX), for all tested bacteria in the model, except for *F. nucleatum* (Table 1 and Table 2). Xanthohumol showed also higher reductions than CHX, excluding *S. oralis* and *A. actinomycetemcomitans* (Table 1). However, differences were small in magnitude and not statistically significant (Table 2).

Finally, incubation of the sample in the presence of 2.5% dimethyl sulfoxide (DMSO) showed no effect on the number of viable cells (Table 1 and Table 2). Thus, a bactericidal effect of this solvent at the concentration used to dissolve xanthohumol and curcumin was ruled out.

#### 2.2.2. Confocal Laser Scanning Microscopy (CLSM) Analysis 

**Figure 3:**
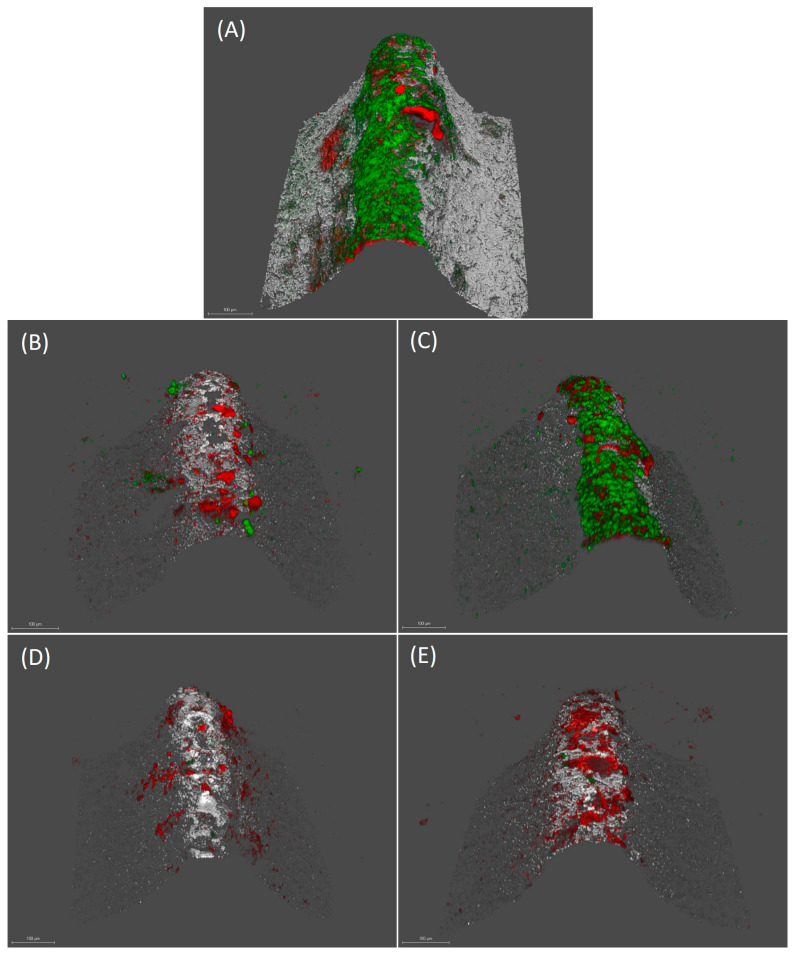
Images obtained by confocal laser scanning microscopy (CLSM) at 72 h over implants treated with phosphate buffer saline (PBS) (**A**), 0.2% chlorhexidine (CHX) (**B**), 2.5% dimethyl sulfoxide (DMSO) (**C**), µM xanthohumol 100 (**D**) and 5 mM curcumin (**E**) (scale bar = 100 µm). LIVE/DEAD^®^ BackLight Kit was used. Live bacteria (green), dead bacteria (red) and implant surface (white) can be differentiated.

Figure 3 shows the mature biofilms on the implants after the respective decontamination treatments, using the tested substances and the controls. The biofilm formed after 72 h showed a live/dead cell ratio of 0.95 (standard deviation (SD) = 0.47) and a bacterial biomass of 13.91 µm^3^/µm^2^ (SD = 10.00). Incubation of the implant with DMSO did not affect either the cell viability (live/dead ratio 0.85 (SD = 0.42)) or the bacterial density of the biofilm (10.73 µm^3^/µm^2^ (SD = 8.81)) (Figure 3a,b).

Decontamination with xanthohumol induced a decrease in biofilm viability, with a live/dead cell ratio of 0.079 (SD = 0.069). Similarly, the use of curcumin reduced cell viability to a live/dead ratio of 0.123 (SD = 0.146) (Figure 3c,d). After xanthohumol and curcumin treatments, the bacterial density of the biofilms was reduced to 6.09 (SD = 5.38) and 6.79 µm^3^/µm^2^ (SD = 5.29), respectively.

Incubation in the presence of CHX reduced the live/dead biofilm cell ratio to 0.25 (SD = 0.25) and the bacterial density to 7.04 µm^3^/µm^2^ (SD = 3.86) (Figure 3d). The reductions in the proportion of live/dead cells in the biofilms, after xanthohumol, curcumin and CHX treatments, were statistically significant (*p* < 0.05). In contrast, differences were not significant for bacterial density.

#### 2.2.3. Scanning Electron Microscopy (SEM) Analysis

**Figure 4:**
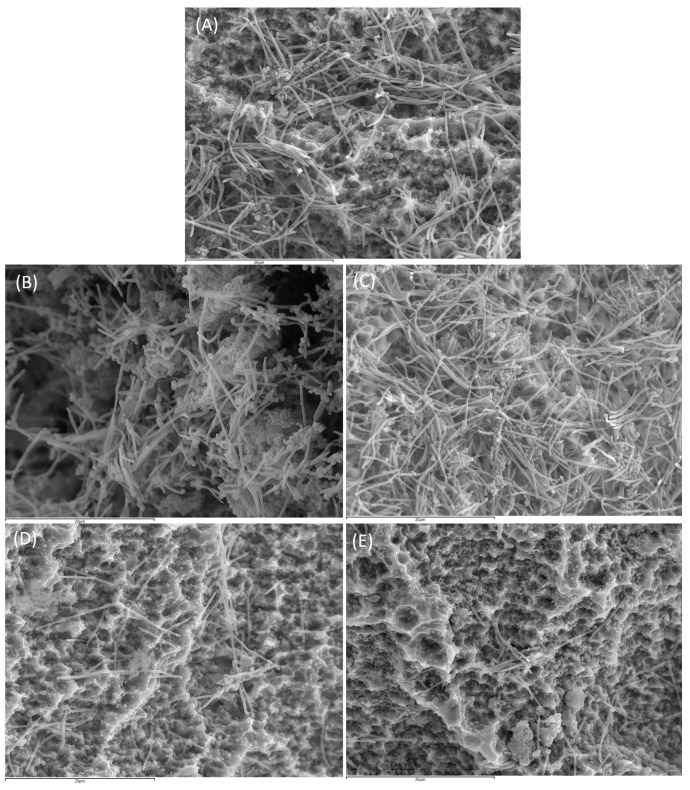
Images obtained by scanning electron microscope (SEM) with 2500× magnification of biofilm developed at 72 h over implants treated with phosphate buffer saline (PBS) (**A**), 0.2% chlorhexidine (CHX) (**B**), 2.5% dimethyl sulfoxide (DMSO) (**C**), 100 µM xanthohumol (**D**) and 5 mM curcumin (**E**) (scale bar = 20 µm).

Figure 4 shows the biofilms on the surface of the implants after treatment with PBS, CHX, DMSO, xanthohumol or curcumin.

Implants treated with PBS and DMSO (Figure 4a,c) showed a high number of cocci and spindle-shaped forms, corresponding to *S. oralis, V. parvula and F. nucleatum*, forming the typical biofilm structure showing the intertwined bacterial communities. Aggregate forms with the presence of *A. actinomycetemcomitans* and, to a lesser extent, coccobacilli corresponding to *P. gingivalis,* could also be observed.

In comparison with the controls, implants treated with xanthohumol, or curcumin (Figure 4d,e) resulted in a clear decrease in the microbial density of the biofilms, with a reduction of fusobacteria in both cases. In these implants, it was also observed the disintegration of the bacterial aggregates, compared with the control biofilms (Figure 4a,c). Figure 4b shows the corresponding reduction in biofilm density after incubation with CHX, although this was less pronounced than in the implants treated with xanthohumol or curcumin.

## 3. Discussion

The results from this in vitro investigation demonstrate that treatment with xanthohumol or curcumin, on mature biofilms developed on dental implant surfaces, significantly reduced the cell viability of the six bacterial strains used in this validated in vitro dynamic biofilm model (*S. oralis, A. naeslundii, V. parvula, F. nucleatum, P. gingivalis* and *A. actinomycetemcomitans*) (Table 1). These reductions were significantly higher, when compared with the negative controls; and similar, even slightly higher for some bacterial species, to those of the positive control used (CHX).

Numerous in vitro studies have evaluated the effect of well-known antibacterial agents, such as CHX (0.12%/0.2%), chloramine (0.1%), citric acid, saline, sodium fluoride (0.055%), stannous fluoride, tetracycline, hydrogen peroxide (H_2_O_2_) (3%), citric acid and hydrogen peroxide, for decontaminating dental implant surfaces using different in vitro models [45,46,47,48,49]. These studies, although some evaluate multiple species and saliva from patients [50], have shown heterogeneous results, since the growing conditions of these models differ to a great extent from those found within the oral cavity [51]. 

To overcome these difficulties, our research group developed a static multi-species in vitro biofilm model on implant surfaces, where the biofilms could be compared among different surface topographies [52,53]. This static model lacked the environmental conditions mimicking the oral cavity and the effect of the flow rate [54], hence the possible translation of the efficacy demonstrated in those studies by the different implant surface decontamination methods could be questioned [23].

In the present study, a dynamic biofilm model has been used which, apart from better reproducing the environmental conditions from the oral cavity, uses *Robbins* devices placed in series which ensures a constant fluid flow under anaerobiosis, not affecting the bacterial viability and allowing the assessment of multiple samples using different experimental techniques (quantitative polymerase chain reaction (qPCR), confocal laser scanning microscopy (CLSM), scanning electron microscopy (SEM)) [22,23].

Chlorhexidine (CHX) was selected as the positive control, since this antibacterial agent has shown well documented efficacy, against both Gram-negative and Gram-positive bacteria, as well as for fungi and some viruses [55,56]. This effect is due to the cationic properties of this molecule, that enables its adsorption to both hard and soft tissues, being released over time (substantivity). However, despite these clear antimicrobial effects, clinical studies on CHX used as a mouth rinse in patients with peri-implant mucositis have not resulted in significant reductions in peri-implant tissue inflammation, when compared with a placebo rinse [57]. Moreover, CHX has side effects such as taste alterations and tooth staining [58], as well as other less frequent complaints as burning or anesthetized sensation, hypersensitivity, and increase in calculus accumulation [59]. Also in vitro studies have shown cytotoxicity, mainly against osteoblasts [60,61,62,63]. Considering these results and the potential problem of the emergence of microbial resistance, mainly with the use of antibiotics [24,25,26], there is a growing interest in studying substances of plant origin (phytochemicals) with demonstrated antimicrobial effect.

In this regard, curcumin and xanthohumol were selected in the present study, due to the scientific evidence regarding the antimicrobial effects of both extracts, when evaluated in vitro and when assessed in clinical studies in a variety of indications. 

The antibacterial activity of curcumin was firstly published by *Nature* in 1949 [64]. In 1974, the in vitro efficacy of curcumin against Gram-positive cocci (*Staphylococcus aureus*, *Staphylococcus epidermidis*, *Streptococcus pyogenes*, *Micrococcus tetragenus*, *Micrococcus luteus*), spore-forming bacilli (*Bacillus* and *Clostridium* species), some Gram-negative bacteria (*Acinetobacter lwoffii, Alcaligenes faecalis*), and fungi (e.g., *Candida stellatoidea, Cryptococcus neoformans, Microsporum gypseum, Saccharomyces cerevisiae, Scopulariopsis brevicaulis*) was also demonstrated [65]. Most recent studies have further confirmed its strong antimicrobial potential, despite its poor solubility in water, low bioavailability and pharmacokinetic profile [39]. In fact, curcumin has shown efficacy in reducing *Streptococcus mutans* mono-species biofilms generated in artificial oral environments [66], and *S. mutans* biofilm formation in in vitro dental models [67].

Although curcumin-based mouthwashes have been introduced as anti-plaque and anti-gingivitis agents [68], and clinical trials have evaluated its efficacy [69,70,71,72,73], its use in the treatment of peri-implant diseases has not yet been reported, although there is one study evaluating the impact of curcumin nanocrystals on *P. gingivalis* strains isolated from patients with implant failure [74].

To explain this antimicrobial effect, experimental studies have shown that curcumin has inhibitory activity on the growth of many bacterial species as *Eschericha coli, Pseudomonas aeruginosa, S. aureus, Bacillus subtilis* and *Enterococcus faecalis* [27] at concentrations similar to those used in the present study. This effect has been explained by the ability of this molecule to inhibit bacterial Quorum-Sensing and to unbundle already formed biofilms [27]. Similarly, curcumin has shown to inhibit SortaseA activity, which is involved in the maintenance of the polysaccharides structure [75]. These possible mechanisms have been corroborated in the present study, demonstrating a clear antibiofilm effect assessed with both CLSM and SEM. This antimicrobial activity of curcumin extracts has been shown to be concentration dependent, since studies demonstrating a potent bactericidal effect on *S. oralis, F. nucleatum* and *P. gingivalis* at concentrations of 1 mM in planktonic growth models had a reduced activity against the same strains in biofilm state [76]. 

In the present work, a concentration of 5 mM was selected, demonstrating a clear bactericidal effect on the six bacteria tested forming a mature biofilm. This effect was similar, and even slightly higher for some bacterial species used in the model, to the one exerted by CHX, but without any evidence of side effects, although both curcumin and xanthohumol are plant substrates with strong staining properties that may limit their clinical use. In clinical studies, however, curcumin has shown to be safe and well-tolerated by the patients [77], and in experimental studies it has not shown cytotoxicity [76]. On the other hand, its anti-inflammatory effect makes curcumin a promising agent for its therapeutic use in the treatment of peri-implant diseases [78].

The first reports on the use of xanthohumol as a mouth rinse to suppress dental plaque regrowth demonstrated significant reductions of *S. mutans* in plaque samples with no unexpected side-effects [79]. In fact, its minimal resorption in the intestine, allows for its administration in large doses with minimal side effects, and has the potential to be used in combination with other molecules [80,81]. Interestingly, when used with chitosan as carrier, it has shown a synergistic effect significantly reducing biofilms formed by *S. aureus* and, to a lesser extent, *P. aeruginosa*, as well as different strains of *Candida* [82].

Apart from the bactericidal effect demonstrated by this extract in the present study, its antibiofilm effect could be related to its demonstrated ability to inhibit lipid metabolism, thus altering hydrophobicity of the bacterial cell wall [83], and also affecting cell adhesion and hence, the structure of the biofilm [40,84]. Xanthohumol has also shown inhibition of the Quorum-Sensing [85]. 

Its potential use in the prevention and treatment of peri-implant diseases could be reinforced by its anti-inflammatory effect related to the enhanced expression of the ERK and AKT genes involved in the inflammatory response [86]. Although in vitro studies have shown that xanthohumol may have adverse cytotoxic effects, these have been demonstrated at concentrations above 100 µM [87], which are above those used in this investigation.

Although the present study is the first to demonstrate the antibiofilm effect of curcumin and xanthohumol extracts on contaminated implant surfaces, using a validated multi-species in vitro dynamic biofilm model, the obtained results should be interpreted with caution considering the limitations of this experimental investigation. Firstly, despite the effort to mimic the oral cavity conditions using this dynamic in vitro model, these cannot be fully reproduced in vitro. Furthermore, only six bacterial species were used, in comparison with the thousands present in naturally occurring subgingival biofilms. Regarding the extracts analyzed, their commercial formulation entails their dissolution in DMSO, which needed its introduction as a control to rule out that the potential antiseptic effect of these agents was due to DMSO rather than to the extracts tested. Furthermore, the experimental results found in the literature nowadays do not allow us to accurately state the mechanism of biological activity of these substances, so future studies could be relevant to characterize it. An additional limitation is the high inter-experiment variability that occurs when working in vitro with live bacteria, what may limit the precision of the quantitative data.

In spite of the limitations of the study, the reported results of the two extracts showed consisting antibacterial and antibiofilm effects, with the assessment methods used (qPCR, CLSM and SEM) and in comparison with the positive control (CHX), what indicates that this investigation may be a primary step in the research process to identify curcumin and xanthohumol extracts as possible candidate molecules for their preventive, therapeutic and maintenance use in peri-implant diseases, although this potential should be further investigated in studies with a higher level of scientific evidence [77].

## 4. Materials and Methods

### 4.1. Curcumin and Xanthohumol

Curcumin from *Curcuma longa* L. (turmeric) was obtained from Sigma-ALDRICH^®^ (Steinheim, Germany) and xanthohumol from NATECO^®^ GmbH & Co (Wolnzach, Germany). These extracts were resuspended in 2.5% (*v*/*v*) DMSO (AppliChen GinbH, Darmsstadt, Germany).

### 4.2. Bacterial Strains and Culture Conditions

*S. oralis* CECT 907T, *V. parvula* NCTC 11810, *A. naeslundii* ATCC 19039, *F. nucleatum* DMSZ 20482, *P. gingivalis* ATCC 33277 and *A. actinomycetemcomitans* DSMZ 8324 were grown on Blood Agar plates (Blood Agar Oxoid No 2; Oxoid, Basingstoke, UK), supplemented with 5% (*v*/*v*) sterile horse blood (Oxoid), 5.0 mg/L haemin (Sigma, St. Louis, MO, USA) and 1.0 mg/L menadione (Merck, Darmstadt, Germany) at 37 °C for 48 h under anaerobic conditions (10% H_2_, 10% CO_2_ and N_2_ balance). 

### 4.3. Antibacterial Effect of Curcumin and Xanthohumol against Planktonic Bacteria 

To select the optimal concentrations of xanthohumol and curcumin for their use in the dynamic biofilm model, MICs and MBCs of both substances against the six selected bacterial strains were determined [88]. 

First, isolated colonies from each strain were grown in protein-enriched medium containing brain-heart infusion (BHI) (Becton, Dickinson and Company, Sparks, MD 21152, USA) supplemented with 2.5 g/L mucin (Oxoid), 1.0 g/L yeast extract (Oxoid), 0.1 g/L cysteine (Sigma), 2.0 g/L sodium bicarbonate (Merck), 5.0 mg/L hemin (Sigma), 1.0 mg/L menadione (Merck) and 0.25% (*v*/*v*) glutamic acid (Sigma) at 37 °C under anaerobic conditions (10% H_2_, 10% CO_2_, and balance N_2_). 

The exponential growth phase was detected by spectrophotometry, the cultures always being below an optical density (OD_550nm_) of 1.2. Once this exponential growth was reached, 200 μL from each bacteria inoculum were transferred to a 24-well microplate reaching a final concentration of 10^6^ CFUs mL^−1^, as previously characterized by our group [89]. Then, xanthohumol (NATECO^®^ GmbH & Co, Wolnzach, Germany) at final concentrations of 10, 20, 50, 100 and 200 µM and curcumin (Sigma-Aldrich^®^, Steinheim, Germany) at final concentrations of 10, 100, 500, 1000 and 5000 µM were added, also using PBS as a negative control. These microplates were incubated for 24 h at 37 °C in anaerobic conditions. MIC and MBC were determined on Blood Agar media plates (Blood Agar Oxoid No 2; Oxoid, Basingstoke, UK), supplemented with 5% (*v*/*v*) sterile horse blood (Oxoid), 5.0 mg/L hemin (Sigma, St. Louis, MO, USA) and 1.0 mg/L menadione (Merck, Darmstadt, Germany), on which 100 µl of each suspension were added. The lowest concentrations of xanthohumol and curcumin extracts showing visible inhibition of bacterial growth were considered as the MICs, while the lowest concentrations of the same extracts showing no bacterial growth after incubation for 72 h were considered as the MBCs. All experiments were performed in triplicate with appropriate controls.

### 4.4. In Vitro Multi-species Dynamic Biofilm Model

**Figure 5:**
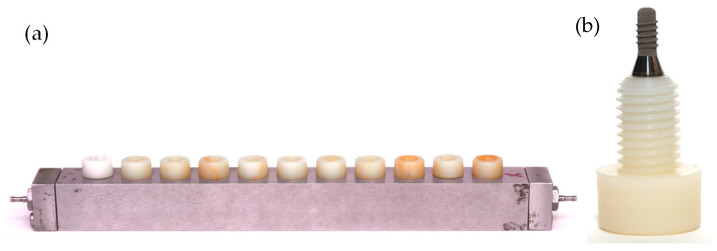
(**a**) *Robbins* device hosting the (**b**) nylon anchoring screws that carry the implants.

Sterile units of commercially available, Straumann^®^ Tissue Level Standard dental implants (Institute Straumann AG, Basel, Switzerland), of 8 mm in length and 3.3 mm in diameter, containing the patented sand-blasted and acid-etched moderately rough surface (SLA), were fixed in customized *Robbins* devices (Figure 5).

**Figure 6:**
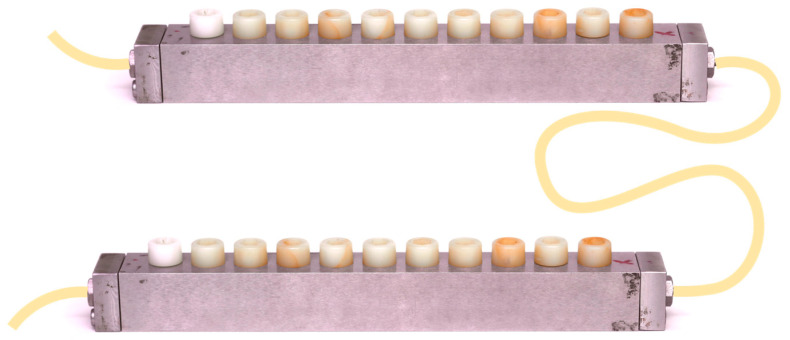
Schematic representation of *Robbins* devices placed in series hosting the implants.

A multi-species dynamic in vitro biofilm model was developed, as previously described by Ribeiro-Vidal et al. (2019) [22,23], with the modification of placing the *Robbins* devices (Figure 6) in series.

Briefly, pure bacterial cultures, obtained from each bacterial species, were grown under anaerobic conditions in protein-rich medium containing modified BHI (Becton, Dickinson and Company, Franklin Lakes, NJ, USA) supplemented with 2.5 g/L mucin (Oxoid), 1.0 g/L yeast extract (Oxoid), 0.1 g/L cysteine (Sigma), 2.0 g/L sodium bicarbonate (Merck), 5.0 mg/L hemin (Sigma), 1.0 mg/L menadione (Merck) and 0.25% (*v*/*v*) glutamic acid (Sigma). After 24 h of incubation, bacterial growth was measured spectrophotometrically (OD_550nm_) to prepare a bacterial mixture containing 10^6^ CFU mL^−1^ for each of the six strains. 

**Figure 7:**
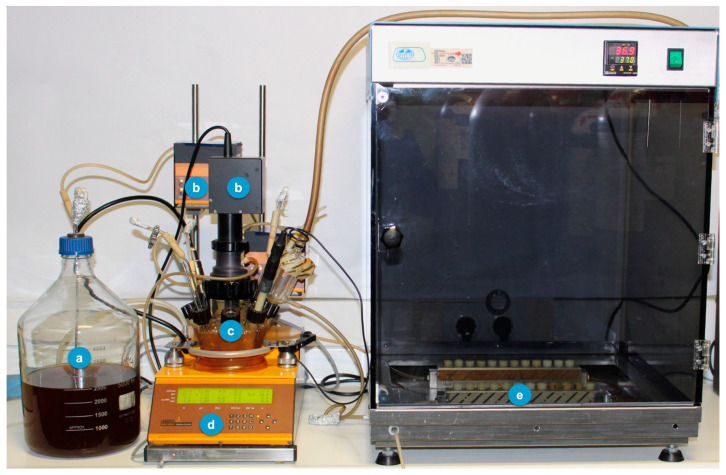
Modified model to generate biofilm over implants with the *Robbins* devices in series. (**a**) Culture medium—modified BHI; (**b**) Peristaltic pumps; (**c**) Incubation recipient; (**d**) Bioreactor (temperature control, pH, pO_2_, agitation and weight); (**e**) *Robbins* devices in series hosting the implants.

This dynamic in vitro biofilm model consists of a sterile vessel (Figure 7a), where the bacterial inoculum is placed within the modified BHI liquid culture medium, which is then pumped by a peristaltic pump, at constant pressure (Figure 7b), to a bioreactor (Lambda Minifor© bioreactor, LAMBDA Laboratory Instruments, Sihlbruggstrasse, Switzerland), that maintains the culture medium at 37 °C, pH 7.2 and constant anaerobic conditions (10% H_2_, 10% CO_2_, and balance N_2_), maintained by constant flow of the gas mixture (Figure 7c,d), thus mimicking the oral cavity environment. This culture medium is then continuously transferred (30 mL/h) through another peristaltic pump (Figure 7b) to the two *Robbins* devices serially placed (Figure 6), within a laboratory stove to keep controlled temperature (37 ºC) and containing the sterile implants under anaerobic conditions (Figure 7e).

During the experimental period, the implants mounted on the *Robbins* devices in series (Figure 5 and Figure 6) have their surfaces within the channel where the bacterial mixture flows, thus allowing the formation of a 72-h mature biofilm over the whole surface of the implants. 

### 4.5. Decontamination Process

**Figure 8:**
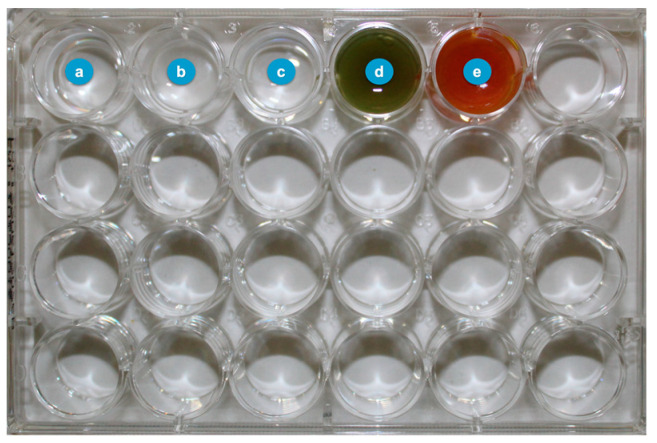
Microplate wells with the decontamination groups (**a**) phosphate-buffered saline (PBS), (**b**) chlorhexidine (CHX), (**c**) dimethyl sulfoxide (DMSO), (**d**) xanthohumol and (**e**) curcumin.

After this incubation period, the implants containing on their surfaces the 72-h biofilms were removed from the *Robbins* devices and placed in microplate wells containing 1 mL of curcumin (5 mM) or 1 mL of xanthohumol (100 µM) for 60 seconds (Figure 8), resuspended both in 2.5% (*v*/*v*) DMSO (AppliChen GinbH, Darmsstadt, Germany). As negative controls, 1 mL of phosphate-buffered saline (PBS) or 1 mL of 2.5% (*v*/*v*) DMSO (to rule out the bactericidal effect of this solvent), were used. As positive control, 1 mL of 0.2% (*v*/*v*) CHX (Sigma-Aldrich, Steinheim, Germany) was employed. 

For each experimental condition, the protocol was repeated three times in three independent sets of experiments. Thus, analysing a total of nine implants (*n* = 9) with qPCR, six implants (*n* = 6) with CLSM and six implants (*n* = 6) with SEM, for each condition.

### 4.6. qPCR Analysis to Evaluate the Antimicrobial Efficacy 

After the referred decontamination process, the implants were sequentially rinsed three times for 10 seconds in 2 mL of PBS to remove bacteria not attached to the biofilm and then, vortexed for 2 min at room temperature in vials containing 1 mL of PBS to disaggregate the bacteria within biofilm. These resulting bacteria were then treated with propidium monoazide (PMA) (Biotium Inc., Hayword, CA, USA) to discriminate DNA from live and dead bacteria [90]. In brief, the vials containing the samples with 100 µM PMA were incubated for 10 min in the dark at 4 °C, followed by incubation in the PMA-Lite LED Photolysis Device (Biotium Inc.) for 20 min at room temperature. After PMA photo-induced DNA hybridization, the samples were centrifuged at 12,000 rpm for 3 min at room temperature and, from the pellet obtained, DNA was extracted following the manufacturer’s instructions from a commercial kit (MolYsisComplete5 Kit; Molzym GmbH & CoKG (Bremen, Germany)). 

To detect and quantify each DNA from the bacterial species isolated in the model, qPCR assays were used. qPCR amplification followed the protocol previously optimized by our research group, using primers and probes targeted against 16S *rRNA* gene ((Life Technologies Invitrogen (Carlsbad, CA, USA), Applied Biosystems (Carlsbad, CA, USA) and Roche (Roche Diagnostic GmbH, Mannheim, Germany)) [91]. These specific primers and probes targeted the 16S rRNA gene of each bacterium at optimal concentrations (*S. oralis*: 900, 900 and 300 nM; *A. naeslundii* and *P. gingivalis* 300, 300 and 300 nM; *V. parvula*: 750, 750 and 400 nM; *A. actinomycetemcomitans:* 300, 300 and 200 nM *and F. nucleatum*: 600, 600 and 300 nM). Each DNA sample was analysed in duplicate. Amplification was performed in a total reaction volume of 10 µL of sterile water (Roche) containing 5 µL of 2X master mix (LC 480 Probes Master, Roche), the corresponding concentrations of the primers and probe and 2.5 µl of the sample. As a negative control, 2.5 µL of sterile water (no template control, NTC) was used. This reaction was prepared in (FramStar 480) natural frame 386-well plates (4titude; The North Barn, Damphurst Lane, UK), sealed by QPCR Adhesive Clear Seals (4titude) and after an initial amplification cycle at 95 °C for 10 min, 40 cycles were performed at 95 °C for 15 seconds and at 60 °C for 1 minute, using a thermal cycler (LightCycler^®^ 480 II Roche Diagnostic GmbH, Mannheim, Germany). Quantification of viable cells by qPCR was based on standard curves correlating Cq values with known CFUs mL^−1^ and it was automatically generated by the software (LC 480 Software 1.5; Roche Diagnostic GmbH; Mannheim, Germany).

### 4.7. CLSM Analysis to Evaluate the Antibiofilm Effect

A laser scanning confocal microscope (Leica SP9, Mannheim, Germany—Centre of Microscopy in the National Centre for Scientific Research (CSIC), Moncloa Campus, University Complutense of Madrid) was used to non-invasively analyse the resulting biofilms, after the decontamination process, and to quantify the biofilm bacterial biomass. Firstly, the implants retrieved from the *Robbins* devices were gently rinsed three times sequentially in 2 mL of sterile PBS (10 seconds of immersion per wash) to remove any remnants of the non-binding bacteria. Then, three separate, representative locations were selected on the implants based on the presence of fully hydrated biofilms (presence of columns or towers of bacterial communities, identified in the confocal field of vision). Subsequently, the biofilms were stained with the LIVE/DEAD^®^ BacLightTM Bacterial Viability Kit solution (Molecular Probes B.V., Leiden, The Netherlands) consisting of Syto9 and Propidium Iodide in a 1:1 ratio for 9 ± 1 min at room temperature, to achieve the optimal fluorescence signal at the corresponding wavelengths (Syto9: 515–530 nm, propidium iodide (PI): >600 nm) 

Finally, a series of scans (xyz) of 1 µm thickness (8 bits, 512 × 512 pixels) were obtained to collect image stacks that were analysed with the imaging software associated with the microscope used (LAS X Leica^®^, Mannheim, Germany). The biomass of live and dead cells was calculated in micrometres^3^/micrometres^2^ (µm^3^/µm^2^) using the COMSTAT software (www.comstat.dk) on the previously collected image films.

### 4.8. SEM Analysis to Evaluate the Morphology of the Biofilms on the Implant Surfaces

For SEM analysis, the biofilms formed on the implants were retrieved from the *Robbins* devices and the decontamination process took place. Afterwards, they were firstly fixed for 4 h at 4ºC with a fixative solution of 4% paraformaldehyde (Panreac Química, Barcelona, Spain) and 2.5% glutaraldehyde (Panreac Química, Barcelona, Spain) after being rinsed three times with 2 mL of PBS (10-s immersion per wash). Subsequently, the samples, after being again rinsed with PBS and sterile water (10-s immersion per wash), were dehydrated using a series of graded ethanol solutions (30, 50, 70, 80, 90 and 100%) with an immersion time of 10 min in each case. Finally, the samples were brought to critical drying point and coated with gold via sputtering. The resulting samples were analysed at the National Centre of Electron Microscopy (ICTS, University Complutense of Madrid, Madrid, Spain) with a JSM 6400 scanning electron microscope equipped with a backscattered electron detector at an image resolution of 25 kV (JSM6400, JEOL, Tokyo, Japan). 

### 4.9. Data Analysis

The primary outcome variable was the counts of viable bacteria present in the biofilm of each implant, measured by qPCR, for each tested bacterial species: *S. oralis*, *A. naeslundii*, *V. parvula*, *A. actinomycetemcomitans*, *P. gingivalis* and *F. nucleatum*. This outcome was expressed as means and SDs of CFUs mL^−1^. From the mean values of each group, the percentage of reduction was calculated for xanthohumol, curcumin, DMSO or CHX, when compared to PBS negative control value [92].

As secondary outcome variables, from CLSM analysis, the resulting microbial biomass was expressed in micrometres^3^/micrometres^2^ (µm^3^/µm^2^), from which live/dead ratios were determined in each case.

The Shapiro–Wilk goodness-of-fit test was used to assess for normality in the data distribution. Data were expressed as means and SD. Comparisons were tested applying ANOVA tests with *post-hoc* Bonferroni corrections, both in the primary and secondary variables. Statistically significant differences were considered for *p*-values <0.05. A software package (IBM SPSS Statistics 27.0, IBM Corporation, Armonk, NY, USA) was used for all data analyses.

## 5. Conclusions

Curcumin and xanthohumol extracts have demonstrated an antibacterial effect against six bacterial species in a validated in vitro dynamic biofilm model. Specifically, these extracts (curcumin at 5 mM and xanthohumol at 100 μM) resulted in statistically significant reductions in live CFUs mL^−1^ of *S. oralis*, *A. naeslundii*, *V. parvula*, *F. nucleatum*, *P. gingivalis* and *A. actinomycetemcomitans* after 60 seconds of exposure. Furthermore, the two extracts showed antibiofilm effect, as assessed by both CLSM and SEM.

## Figures and Tables

**Table 1:** Antibacterial effects of curcumin (CUR) and xanthohumol (XN), as observed in the mean number of viable bacteria counts [colony forming units per mL (CFUs) mL^−1^, determined by qPCR], evaluated in an in vitro multi-species dynamic biofilm model. Data are expressed as mean and standard deviation (SD). Differences are considered statistically significant at *p* < 0.05.

Bacterial Species	Treatments	Mean (SD)	Global *p*	% Reduction of Viable Counts, as Compared with PBS
***S. oralis***	PBS	7.00 × 10^5^ (4.00 × 10^5^)	<0.000	Reference
	XN	1.77 × 10^4^ (1.71 × 10^4^)		97.47
	CUR	7.74 × 10^3^ (6.10 × 10^5^)		98.89
	DMSO	8.54 × 10^5^ (1.23 × 10^7^)		NA
	CHX	1.36 × 10^4^ (1.39 × 10^4^)		98.05
***A. naeslundii***	PBS	1.56 × 10^7^ (1.52 × 10^7^)	0.000	Reference
	XN	5.25 × 10^5^ (4.34 × 10^5^)		96.64
	CUR	3.28 × 10^5^ (6.90 × 10^5^)		97.90
	DMSO	1.08 × 10^7^ (9.65 × 10^6^)		30.67
	CHX	9.36 × 10^5^ (9.67 × 10^5^)		94.01
***V. parvula***	PBS	8.81 × 10^7^ (6.99 × 10^7^)	<0.000	Reference
	XN	1.31 × 10^6^ (1.25 × 10^6^)		98.52
	CUR	2.59 × 10^5^ (2.72 × 10^5^)		99.71
	DMSO	6.80 × 10^7^ (5.36 × 10^7^)		22.74
	CHX	2.91 × 10^6^ (6.35 × 10^6^)		96.70
***F. nucleatum***	PBS	7.70 × 10^5^ (4.94 × 10^5^)	<0.000	Reference
	XN	3.16 × 10^4^ (4.12 × 10^4^)		95.90
	CUR	5.61 × 10^4^ (1.13 × 10^5^)		92.71
	DMSO	6.58 × 10^5^ (4.03 × 10^5^)		14.51
	CHX	4.57 × 10^4^ (8.37 × 10^4^)		94.06
***P. gingivalis***	PBS	1.38 × 10^6^ (5.26 × 10^5^)	<0.000	Reference
	XN	1.11 × 10^4^ (5.80 × 10^3^)		99.20
	CUR	3.87 × 10^3^ (2.47 × 10^3^)		99.72
	DMSO	1.41 × 10^6^ (1.01 × 10^6^)		NA
	CHX	2.51 × 10^4^ (2.60 × 10^4^)		98.18
***A. actinomycetemcomitans***	PBS	4.81 × 10^5^ (4.46 × 10^5^)	0.001	Reference
	XN	1.62 × 10^4^ (1.34 × 10^4^)		96.64
	CUR	5.82 × 10^3^ (4.33 × 10^3^)		98.79
	DMSO	4.76 × 10^5^ (5.73 × 10^5^)		1.06
	CHX	1.38 × 10^4^ (9.06 × 10^3^)		97.12

PBS: phosphate buffer saline; CUR: 5 mM curcumin; XN: 100 μM xanthohumol; DMSO: Dimethyl sulfoxide; CHX: 0.2% chlorhexidine; NA: not applicable.

**Table 2:** Comparisons between experimental treatments and the controls used as observed in the mean number of viable bacteria counts (colony forming units per mL (CFUs) mL^−1^, determined by qPCR) evaluated in an in vitro multi-species dynamic biofilm model. Differences are considered statistically significant at *p* < 0.05.

Bacterial Species	Comparisons	Mean Difference	95% Confidence Interval for Difference		*Post-Hoc p*
			Lower Bound	Upper Bound	
***S. oralis***	PBS-CHX	6.87 × 10^5^	2.29 × 10^5^	1.14 × 10^6^	0.001
	PBS-XN	6.83 × 10^5^	2.25 × 10^5^	1.14 × 10^6^	0.001
	PBS-CUR	6.93 × 10^5^	2.35 × 10^5^	1.15 × 10^6^	0.001
	PBS-DMSO	−1.53 × 10^5^	−6.10 × 10^5^	3.03 × 10^5^	1.000
	CHX-XN	−4.10 × 10^3^	−4.61 × 10^5^	4.53 × 10^5^	1.000
	CHX-CUR	5.89 × 10^3^	−4.51 × 10^5^	4.63 × 10^5^	1.000
	CHX-DMSO	−8.40 × 10^5^	−1.29 × 10^6^	−3.83 × 10^5^	<0.001
	XN-CUR	9.99 × 10^3^	−4.47 × 10^5^	4.67 × 10^5^	1.000
	XN-DMSO	−8.36 × 10^5^	−1.29 × 10^6^	−3.79 × 10^5^	<0.001
	CUR-DMSO	−8.46 × 10^5^	−1.30 × 10^6^	−3.89 × 10^5^	<0.001
***A. naeslundii***	PBS-CHX	1.47 × 10^7^	3.39 × 10^6^	2.60 × 10^7^	0.004
	PBS-XN	1.51 × 10^7^	3.80 × 10^6^	2.64 × 10^7^	0.003
	PBS-CUR	1.53 × 10^7^	4.00 × 10^6^	2.66 × 10^7^	0.002
	PBS-DMSO	4.79 × 10^6^	−6,51 × 10^6^	1.61 × 10^7^	1.000
	CHX-XN	4.11 × 10^5^	−1.09 × 10^7^	1.17 × 10^7^	1.000
	CHX-CUR	6.07 × 10^5^	−1.07 × 10^7^	1.19 × 10^7^	1.000
	CHX-DMSO	−9.90 × 10^6^	−2.12 × 10^7^	1.40 × 10^6^	0.129
	XN-CUR	1.96 × 10^5^	−1.11 × 10^7^	1.15 × 10^7^	1.000
	XN-DMSO	−1.03 × 10^7^	−2.16 × 10^7^	9.94 × 10^5^	0.099
	CUR-DMSO	−1.05 × 10^7^	−2.18 × 10^7^	7.98 × 10^5^	0.086
***V. parvula***	PBS-CHX	8.52 × 10^7^	2.99 × 10^7^	1.40 × 10^8^	<0.001
	PBS-XN	8.68 × 10^7^	3.15 × 10^7^	1.42 × 10^8^	<0.001
	PBS-CUR	8.78 × 10^7^	3.25 × 10^7^	1.43 × 10^8^	<0.001
	PBS-DMSO	1.97 × 10^7^	−3.55 × 10^7^	7.50 × 10^7^	1.000
	CHX-XN	1.60 × 10^6^	−5.37 × 10^7^	5.69 × 10^7^	1.000
	CHX-CUR	2.65 × 10^6^	−5.27 × 10^7^	5.80 × 10^7^	1.000
	CHX-DMSO	−6.54 × 10^7^	−1.20 × 10^8^	−1.01 × 10^7^	0.011
	XN-CUR	1.05 × 10^6^	−5.43 × 10^7^	5.63 × 10^7^	1.000
	XN-DMSO	−6.70 × 10^7^	−1.22 × 10^8^	−1.17 × 10^7^	0.009
	CUR-DMSO	−6.80 × 10^7^	−1.23 × 10^8^	−1.27 × 10^7^	0.007
***F. nucleatum***	PBS-CHX	7.24 × 10^5^	3.14 × 10^5^	1.13 × 10^6^	<0.001
	PBS-XN	7.38 × 10^5^	3.29 × 10^5^	1.15 × 10^6^	<0.001
	PBS-CUR	7.14 × 10^5^	3.04 × 10^5^	1.12 × 10^6^	<0.001
	PBS-DMSO	1.11 × 10^5^	−2.97 × 10^5^	5.21 × 10^5^	1.000
	CHX-XN	1.41 × 10^4^	−3.95 × 10^5^	4.24 × 10^5^	1.000
	CHX-CUR	−1.04 × 10^4^	−4.20 × 10^5^	3.99 × 10^5^	1.000
	CHX-DMSO	−6.12 × 10^5^	−1.02 × 10^6^	−2.02 × 10^5^	0.001
	XN-CUR	−2.45 × 10^4^	−4.34 × 10^5^	3.85 × 10^5^	1.000
	XN-DMSO	−6.26 × 10^5^	−1.03 × 10^6^	−2.16 × 10^5^	<0.001
	CUR-DMSO	−6.01 × 10^5^	−1.01 × 10^6^	−1.92 × 10^5^	0.001
***P. gingivalis***	PBS-CHX	1.35 × 10^6^	6.42 × 10^5^	2.06 × 10^6^	<0.001
	PBS-XN	1.37 × 10^6^	6.55 × 10^5^	2.08 × 10^6^	<0.001
	PBS-CUR	1.37 × 10^6^	6.63 × 10^5^	2.09 × 10^6^	<0.001
	PBS-DMSO	−2.69 × 10^4^	−7.38 × 10^5^	6.84 × 10^5^	1.000
	CHX-XN	1.40 × 10^4^	−6.99 × 10^5^	7.24 × 10^5^	1.000
	CHX-CUR	2.12 × 10^4^	−6.90 × 10^5^	7.33 × 10^5^	1.000
	CHX-DMSO	−1.38 × 10^6^	−2.09 × 10^6^	−6.69 × 10^5^	<0.001
	XN-CUR	7.21 × 10^3^	−7.03 × 10^5^	7.20 × 10^5^	1.000
	XN-DMSO	−1.39 × 10^6^	−2.10 × 10^6^	−6.81 × 10^5^	<0.001
	CUR-DMSO	−1.40 × 10^6^	−2.11 × 10^6^	−6.90 × 10^5^	<0.001
***A. actinomycetemcomitans***	PBS-CHX	4.67 × 10^5^	1.23 × 10^4^	9.22 × 10^5^	0.040
	PBS-XN	4.65 × 10^5^	9.59 × 10^3^	9.20 × 10^5^	0.042
	PBS-CUR	4.75 × 10^5^	2.04 × 10^4^	9.30 × 10^5^	0.035
	PBS-DMSO	5.10 × 10^3^	−4.49 × 10^5^	4.60 × 10^5^	1.000
	CHX-XN	−2.33 × 10^3^	−4.58 × 10^5^	4.52 × 10^5^	1.000
	CHX-CUR	8.02 × 10^3^	−4.47 × 10^5^	4.63 × 10^5^	1.000
	CHX-DMSO	−4.62 × 10^5^	−9.17 × 10^5^	−7.24 × 10^3^	0.044
	XN-CUR	1.03 × 10^4^	−4.44 × 10^5^	4.66 × 10^5^	1.000
	XN-DMSO	−4.59 × 10^5^	−9.14 × 10^5^	−4.48 × 10^3^	0.046
	CUR-DMSO	−4.70 × 10^5^	−9.25 × 10^5^	−1.52 × 10^4^	0.038

PBS: phosphate-buffered saline; CUR: 5 mM curcumin; XN: 100 μM xanthohumol; DMSO: dimethyl sulfoxide; CHX: 0.2% chlorhexidine.
